# Measuring Contamination Levels and Incubation Results of Hatching Eggs Sanitized with Essential Oils

**DOI:** 10.3390/antibiotics14111076

**Published:** 2025-10-26

**Authors:** Vinícius Machado dos Santos, Gabriel da Silva Oliveira, Pedro Henrique Gomes de Sá Santos, Liz de Albuquerque Cerqueira, José Luiz de Paula Rôlo Jivago, Susana Suely Rodrigues Milhomem Paixão, Márcio Botelho de Castro, Concepta McManus

**Affiliations:** 1 Laboratory of Poultry Science, Federal Institute of Brasília—Campus Planaltina, Brasília 73380-900, Brazil; 2 Faculty of Agronomy and Veterinary Medicine, University of Brasília, Brasília 70910-900, Brazil; 3 Institute of Biological Sciences, University of Brasília, Brasília 70910-900, Brazil; 4 Center for Nuclear Energy in Agriculture (CENA), University of São Paulo, São Paulo 13416-000, Brazil

**Keywords:** egg incubation, egg microbiology, natural products, poultry farming, poultry health

## Abstract

**Background/Objectives**: Making sustainable choices and transforming guidelines into effective bacterial control practices for viable and safe hatching eggs is a challenge for many researchers. Gradually, scientific findings are strengthening the case for using antibacterial protocols with essential oils (EOs) for hatching eggs, which could lead to changes in traditional egg sanitization management and stimulate new research. The present study aimed to measure the contamination levels and incubation outcomes of hatching eggs sanitized with *Zingiber officinale* (ZOEO), *Cymbopogon flexuosus* (CFEO), and *Rosmarinus officinalis* (ROEO) essential oils. **Methods**: Hatching eggs from commercial broiler breeders were sanitized with solutions of ZOEO, CFEO, and ROEO prepared in grain alcohol and compared with formaldehyde and non-sanitized eggs. Bacterial contamination, eggshell integrity, incubation parameters, embryonic trachea histology, genotoxicity, and irritation potential were evaluated under commercial conditions. **Results**: It has been demonstrated that these EOs significantly reduce eggshell and yolk sac contamination, promote hatchability rates above 93% with good-quality chicks, and do not cause alterations in the embryonic trachea or potential genetic damage to the chicks. **Conclusions**: ZOEO, CFEO, and ROEO can be recommended as sanitizers for hatching eggs.

## 1. Introduction

The sanitization of hatching eggs is not limited to reducing the microbial load of eggs from nests or litter, such as the reduction in total aerobic mesophilic bacteria (TAMB) before and during incubation. This process can also promote embryonic development, positively influence blood and hormonal constituents, increase hatchability, and improve body weight, weight gain, feed intake, and feed conversion ratio [1,2]. The fumigation of hatching eggs with formaldehyde (FA) is practiced in countries such as Brazil [3], but this practice has been discouraged worldwide. In the 1970s, FA was already employed as an agent to control contamination in eggs intended for incubation [4]. Nevertheless, the first evidence of its embryotoxic effects in poultry was reported in the late 1990s [5]. Only in the late 2000s did a comprehensive review include a critical analysis addressing not only the sanitizing efficacy of FA, but also raising concerns about its toxicological profile in poultry [6]. Furthermore, in the penultimate year of the 1980s, a paper highlighted the risks associated with the toxicity of FA vapor for humans exposed to this compound [7]. Currently, in many countries, including Brazil, the use of natural products is recommended as replacements for FA gas [8,9,10,11,12,13]

One of the current trends is leveraging the power of nature to enhance poultry biosecurity and biosafety. Essential oils (EOs) extracted from plants such as cinnamon (*Cinnamomum cassia* (L.) J. Presl), clove (*Syzygium aromaticum* (L.) Merr. & L.M. Perry), citronella (*Cymbopogon nardus* (L.) Rendle), lemon (*Citrus aurantifolia* (Christm.) Swingle), basil (*Ocimum basilicum* L.), garlic (*Allium sativum* L.), lavender (*Lavandula angustifolia* Mill.), tea tree (*Melaleuca alternifolia* (Maiden & Betche) Cheel), cumin (*Cuminum cyminum* L.), and oregano (*Origanum vulgare* L.) have shown significant potential for improving microbial control practices on eggshells and/or optimizing incubation parameters [14,15,16,17]. These EOs have been integrated into strategic plans aimed at sustainably addressing the challenges of modern poultry farming. The continued evaluation of EOs in the management of hatching egg sanitization is crucial for advancing poultry health. In addition to the oils already studied, many others are available on the global market, offering hundreds of options with diverse properties. Exploring these new oils not only expands knowledge about their applications but also has the potential to establish a long-term resource bank for future poultry production.

*Zingiber officinale* Rosc. (ginger) is an aromatic plant that can reach a height of 66.2 cm, with a trunk diameter of 6.4 mm [18]. The EO content can range from 0.90 to 2.42% [19]. *Zingiber officinale* essential oil (ZOEO) is composed mainly of α-zingiberene, β-sesquiphellandrene, α-curcumene and trans-γ-cadinene [20]. It has been used as an efficient antibacterial enhancer of coatings intended for poultry products [21,22]. *Cymbopogon flexuosus* (Nees ex Steud.) W.Watson (lemongrass) is an aromatic plant that can reach a height of 240 cm, with an EO content that can range from 0.3 to 0.91%. This EO contains citral, D-limonene, linalool, myrcene, methyl eugenol, methyl isoeugenol, α-terpineol, neryl acetate, citronellol, geraniol, geranyl acetate, and elemicin [23]. *Cymbopogon flexuosus* essential oil (CFEO) has been used for antibacterial control in poultry products. For example, its ability to reduce *Salmonella* Heidelberg in chicken meat has been demonstrated [24]. *Rosmarinus officinalis* L. (rosemary) is an aromatic plant that can reach a height of 208 cm, with a stem diameter of 3.0 mm. The EO content can range from 0.51 to 4.56%. This EO is composed chiefly of camphor, α–bisabolol 1,8–cineole, α–pinene, camphene, and β–pinene [25]. Kačániová et al. [26] reported the inhibitory effect of *Rosmarinus officinalis* essential oil (ROEO) on ENT in vacuum-packed chicken breast meat. In other studies, these EOs are effective in controlling different bacteria [27,28,29,30,31,32,33,34,35].

Additional approaches involving EOs from *Cymbopogon*, *Zingiber*, and *Rosmarinus* genera have already demonstrated effectiveness in addressing critical challenges in poultry production, which could lead to increased productivity, reduced mortality in production systems, minimized postcollection losses, and increased food safety [22,36,37,38]. Reported benefits include decreased bacterial load on eggshells, improved embryonic development through enhanced nutrient digestion and absorption, strengthened intestinal integrity and immune function in broilers, mitigation of the negative impacts of necrotic enteritis, optimized feed conversion, and improved meat quality and extended shelf life of poultry products [22,39,40,41,42,43].

Given the beneficial effects of EOs from these genera in poultry production, particularly those derived from the specific species ZOEO, CFEO, and ROEO, and considering the limited number of studies that simultaneously evaluate, under commercial conditions, the antibacterial efficacy of EOs in the sanitization of hatching eggs and their toxicological consequences for the eggshells at the structural level and for poultry at the histological and genetic levels, including assessments using the in vivo Hen’s Egg Test Chorioallantoic Membrane (HET-CAM) model, this study aimed to measure the contamination levels, toxic effects, and incubation performance of hatching eggs sanitized with ZOEO, CFEO, and ROEO.

## 2. Results and Discussion

The mean TAMB on eggshell surfaces before incubation and in yolk sacs differed significantly (*p* < 0.05) among the treatments (Table 1). Both FA and EOs achieved an effective reduction on the eggshell surface, decreasing to <1 log_10_ per plate. However, only the EOs were capable of achieving a 1 log_10_ reduction in the yolk sac. While the FA showed a TAMB similar to that observed in the control group, eggs treated with EOs exhibited significantly lower values. This 1 log_10_ reduction compared to the control highlights the superior antibacterial activity of EOs in reducing bacterial contamination of the yolk sac. The disruption of cellular processes in bacteria, such as energy production, membrane transport, and other metabolism-based regulatory functions, partially explains the antibacterial activity of EOs [44]. In all the treatments, the Enterobacteriaceae (ENT) levels on the eggshell and yolk sac were <1 log_10_ per plate. Similar findings on the effectiveness of EOs in reducing the bacterial load on eggshells and yolk sacs have been previously reported. For example, eggshells of chicken eggs sanitized with *Syzygium aromaticum* EO at concentrations of 0.5, 1, and 2% presented nearly 1.7, 2.3, and 2.6 log_10_ reduction in TAMB one hour after application, respectively [45]. Sanitizers formulated with EOs of *Citrus aurantifolia* (9.38 mg/mL), *Ocimum basilicum* (4.69 mg/mL), and *Allium sativum* (1.17 mg/mL) achieved over a 50% reduction in TAMB and ENT populations in the yolk sacs of 18-day-old chicken embryos [15]. The strong correlation between eggshell contamination and yolk sac contamination in embryos may explain these findings [46], with lower contamination of the eggshell resulting in lower contamination of the yolk sac, which was observed here. Such a response was absent in eggs treated with FA-based sanitization, possibly because of its short-lived antibacterial effect on eggshells [17].

**Figure 1:**
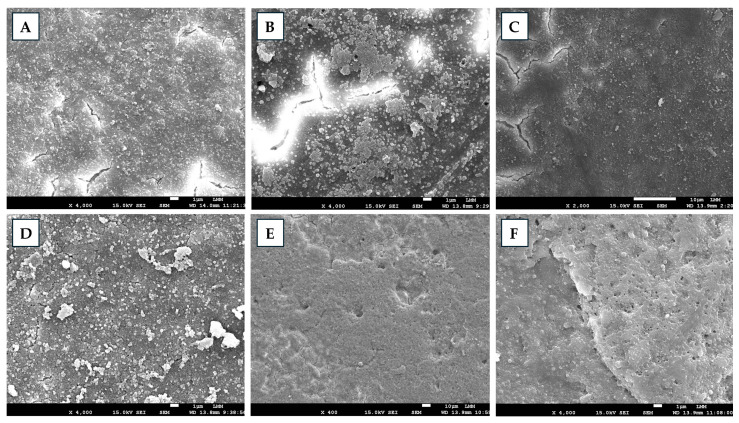
Scanning electron microscopy images of eggshells, untreated and treated with different sanitizers. (**A**) Control; (**B**) grain alcohol—GA; (**C**) formaldehyde—FA; (**D**) *Zingiber officinale* essential oil—ZOEO; (**E**) *Cymbopogon flexuosus* essential oil—CFEO; (**F**) *Rosmarinus officinalis* essential oil—ROEO.

Scanning electron microscopy analysis revealed differences between non-sanitized and sanitized eggshells (Figure 1). The non-sanitized samples exhibited a dense, highly mineralized matrix with irregular surfaces composed of microfissures and natural porosities, as well as interconnected contours and microstructures arranged in a balanced pattern. Sanitization with Grain alcohol (GA) caused strongly irregular surfaces, pronounced discontinuities, and unstable contours, as well as a marked reduction in mineralization. FA induced partial alterations in the matrix and early signs of mineral density loss, whereas the surfaces remained irregular without significant fragmentation. Among the EOs, ZOEO promoted partial matrix modifications, maintaining surface irregularities without severe erosion. Eggshells treated with CFEO presented a more homogeneous outer layer, marked by microdiscontinuities and microfissures, with no signs of advanced wear. ROEO preserved structural density and mineralization, presented slightly irregular surfaces consistent with natural microprojections, an absence of relevant erosion, structural balance, and no increase in critical fissures or pores. ROEO was the sanitizer that most preserved the integrity of the eggshells, whereas GA caused the greatest structural degradation. Oliveira et al. [15] reported that treatments with EOs from *Citrus aurantifolia*, *Ocimum basilicum*, and *Allium sativum* caused moderate to advanced damage to eggshells, likely largely due to the use of GA as a solvent, which alone induced severe surface degradation. In the present study, some alterations attributed to the EOs were also observed, which may, in part, be associated with alcohol. However, the observed behavior differed from that reported previously, as the EOs, particularly ROEO, appeared to mitigate the deleterious effects of the solvent, possibly due to their specific chemical composition. With respect to FA fumigation, Oliveira et al. [15] reported extremely severe damage to eggshells, whereas in the present study, the effects were limited to intermediate alterations, despite the similarity of experimental protocols. It is hypothesized that the interaction of FA with eggshells may be modulated by external factors, such as environmental conditions, or internal factors, such as intrinsic shell characteristics, providing a plausible explanation for the divergence observed between studies.

No significant differences (*p* > 0.05) were observed among treatments for egg weight before setting (EWBS) or egg weight during transfer (EWDT). The alterations in the eggshells caused by the different sanitizers were not sufficient to significantly affect the permeability of the shells between the treatments, as egg weight loss (EWL) did not differ (*p* > 0.05) between them (Table 2). Similarly, Mustafa et al. [47] sprayed a commercial *Lavandula angustifolia* essential oil onto hatching eggs and observed no significant changes in EWL during incubation. Chicks hatched from eggs treated with ZOEO and CFEO showed higher weights (*p* < 0.05) compared to those in the FA group, while all treated egg groups had similar weights (*p* > 0.05) to the control group. This result indicates that FA tended to fail to improve chick weight. In contrast, the EOs, particularly ZOEO and CFEO, tended to enhance chick weight and mitigate the embryotoxic effects of FA. Experiments using EOs on hatching eggs have also been shown to increase the weight of both embryos and newly hatched chicks compared to those treated with FA [16,48]. This finding suggests specific beneficial interactions at various levels, such as microbial, eggshell structural, and/or chemical stress, between the bioactive compounds of the EOs and the developing embryo. Special attention should be given to this aspect, as these effects continue to be reported to date.

Although hatchability (HI) did not differ significantly (*p* > 0.05) among the treatments, there was a tendency for higher HI in eggs treated with EOs than in the control (Table 2). This trend is consistent with previous findings in the literature that attributed the ability of EOs to significantly improve HI, even surpassing the results obtained with conventional synthetic agents or with no treatment at all [16,49]. Notably, this pattern appears to mirror the bacterial load profile observed in the yolk sac. The EOs significantly reduced this bacterial load, a particularly relevant finding considering the well-established negative correlation between yolk sac bacterial load and HI [46]. In addition to the incubation parameters presented in Table 2, the other indicators evaluated in this study, including early (EED), mid (MED), and late embryonic dead (LED), as well as the percentage of contaminated eggs (CE), were not significantly different among the treatments (*p* > 0.05), with overall means of 2.18, 0.25, 3.82, and 0.69%, respectively. The chick quality score (CQS) ranged from 9.70 ± 0.52 for chicks from eggs sanitized with ZOEO to 9.28 ± 0.91 for chicks from the control treatment, with no significant differences (*p* > 0.05) observed among treatments.

The bacteriological and incubation findings showed that lower TAMB on the eggshell and in the yolk sac, especially in eggs sanitized with EOs, tended to have higher HI rates above 93% and heavier chicks at hatch. However, no statistically significant difference in HI was observed among the treatments. Reduced eggshell TAMB but higher levels of this contamination in the yolk sac, as observed in the FA treatment, coincided with slightly lower HI rates and reduced CW at hatch. Maintaining low bacterial counts on eggs during incubation is essential to optimize HI, CW, and overall CQS simultaneously. This perspective allows us to see that the antibacterial efficiency of EOs, in addition to ensuring the sanitary control of hatching eggs, also reflects positively on poultry production outcomes.

**Figure 2:**
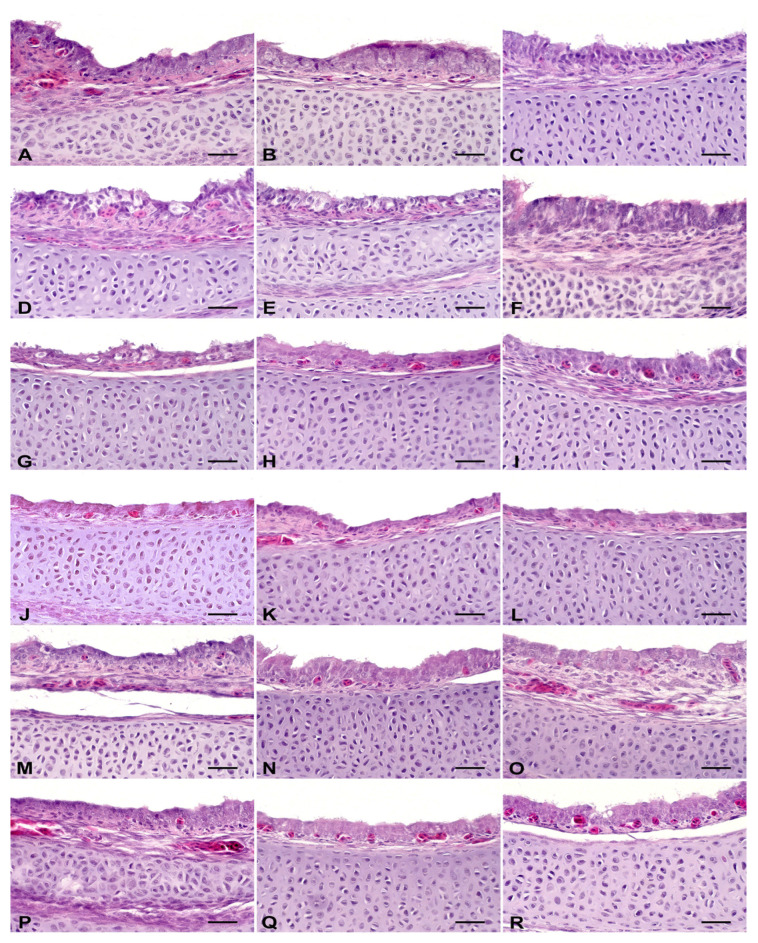
Histological evaluation of the trachea. Hematoxylin and eosin stain, scale bar = 25 µm. Control: (**A**–**C**) showed no morphological changes. Formaldehyde—FA: (**D**) epithelial degeneration; (**E**) goblet cell hyperplasia; (**F**) mononuclear inflammatory infiltrate in the mucosa. Grain alcohol—GA: (**G**) epithelial degeneration; (**H**) no morphological changes; (**I**) no morphological changes. *Zingiber officinale* essential oil—ZOEO: (**J**–**L**)—showed no morphological changes. *Rosmarinus officinalis* essential oil—ROEO: (**M**–**O**)—showed no morphological changes. *Cymbopogon flexuosus* essential oil—CFEO: (**P**–**R**)—showed no morphological changes.

Histological evaluation of the trachea of the embryos revealed that treatment with ZOEO, CFEO, or ROEO did not induce alterations in the analyzed parameters, which were comparable to those of the control group, which also presented no lesions (Figure 2; Table 3). Treatment with GA resulted in mild epithelial degeneration, suggesting a slight irritation effect. FA was the only treatment that caused alterations in three of the four evaluated parameters, indicating a more aggressive effect on tracheal tissue. Similar results have been reported. Tracheal lesions were not observed in 18-day-old chicken embryos from eggs sprayed with EOs of *Citrus aurantifolia*, *Ocimum basilicum* or *Allium sativum* [15] but were reported in embryos from eggs fumigated with FA (5 g/m^3^ or, under the 3× protocol, 42 mL of formalin and 21 g of potassium permanganate per m^3^) during pre-incubation [15,50]. Notably, histopathological lesions in the respiratory tract of chicks, attributed to exposure to FA vapors during fumigation, have been documented for at least two decades [51]. These findings suggest that EOs, in general, may present lower toxicity to embryonic respiratory tissues, particularly the tracheal epithelium, than FA. This difference may be related to the less irritant chemical composition of the oils and to the concentrations used, which may be less aggressive than traditional fumigation.

**Figure 3:**
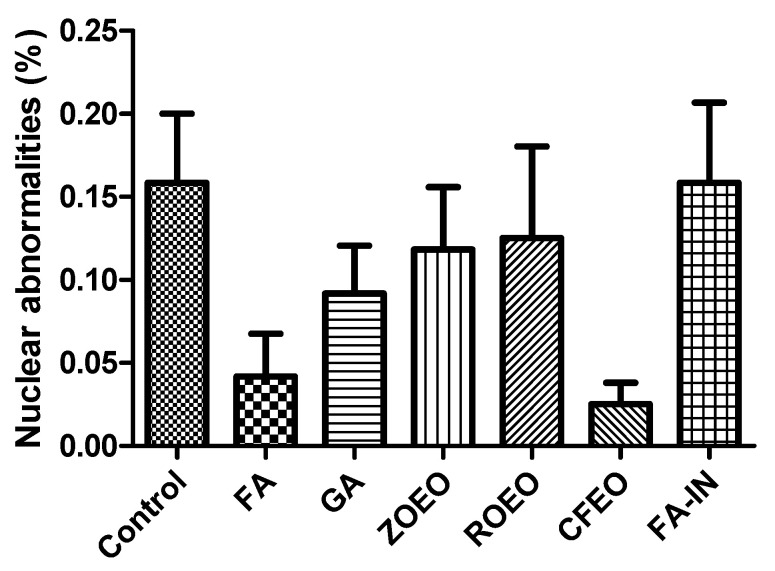
Frequency of nuclear abnormalities in erythrocytes of chicks hatched from sanitized and non-sanitized eggs. Grain alcohol—GA; formaldehyde—FA; *Zingiber officinale* essential oil—ZOEO; *Cymbopogon flexuosus* essential oil—CFEO; *Rosmarinus officinalis* essential oil—ROEO; Formaldehyde applied during incubation—FA-IN.

**Figure 4:**
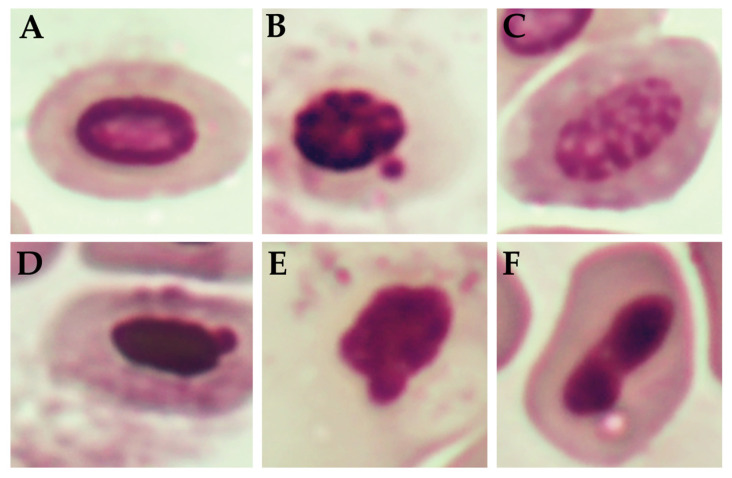
Nuclear defects in erythrocytes of chicks hatched from sanitized and non-sanitized eggs. (**A**) Normal nuclei; (**B**) Micronucleated; (**C**) Apoptotic nuclei; (**D**) Blebbed nuclei; (**E**) Notched nuclei; (**F**) Kidney nuclei.

Figure 3 shows the percentage of nuclear abnormalities observed in the erythrocytes of newly hatched chicks subjected to different treatments. Some of these abnormalities are shown in Figure 4. Although numerical variations were observed among the groups, no statistically significant differences were identified (*p* > 0.05). The control group and the group with the application of FA during incubation (FA-IN) exhibited the highest mean frequency of nuclear alterations, followed by the other groups, which presented varying levels, including FA, GA, ZOEO, CFEO, and ROEO. Even in FA-IN, no significant changes in the percentage of nuclear abnormalities were observed compared with those in the control group. FA absorption by the embryos, confirmed through histological analysis during development, along with the absence of significant genetic damage among treatments, may be related to insufficient exposure to induce such alterations or to the attenuation of genetic lesions through the natural process of hematopoietic renewal, in which immature erythrocytes carrying potential damage may have been replaced by healthy cells before analysis [52].

**Figure 5:**
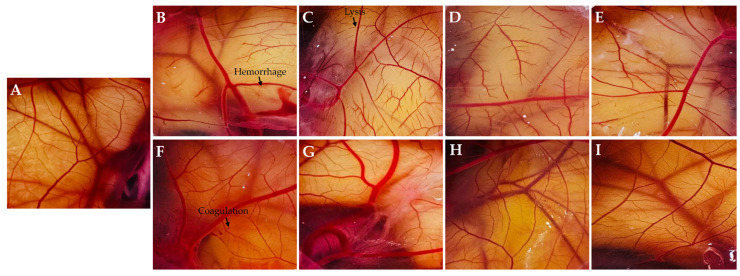
Photographs illustrating the health of chorioallantoic membranes (CAMs) based on the treatment applied. Control (**A**); Grain alcohol (GA) (**B**); *Zingiber officinale* essential oil (ZOEO) (**C**); *Cymbopogon flexuosus* essential oil (CFEO) (**D**); *Rosmarinus officinalis* essential oil (ROEO) (**E**); formaldehyde (FA) (**F**); pure ZOEO (**G**); pure CFEO (**H**); and pure ROEO (**I**).

The characteristic vascular reactions of cell lysis, hemorrhage, and coagulation were assessed using the HET-CAM test (Figure 5). The sanitizer formulated with GA, as well as formulations containing EOs diluted in this vehicle, induced immediate cell lysis, exhibited clear signs of hemorrhage and coagulation throughout the 5 min observation period, and were therefore classified as severe irritants. In contrast, the negative control and treatments with pure EOs did not cause significant vascular changes, except for CFEO, which showed signs of coagulation and occasional hemorrhagic events near the end of the 5 min. This profile is consistent with mild irritation. The results clearly revealed that the toxicity observed in the formulations with diluted EOs was due to the alcohol vehicle, which was also previously reported by Oliveira et al. [15] and Oliveira et al. [53]. EOs, whether in their pure form or diluted in other media, indeed appear to be non-irritating when assessed using the HET-CAM test [54,55]. FA treatment induced vascular responses across all three evaluated parameters within the observation window and was characterized as a moderate irritant sanitizer.

It is important to reinforce the isolated effects of 93.8% GA used as a solvent. The EOs were diluted in GA as a strategy to evaluate whether the alcohol itself, when used alone, presented significant adverse effects on hatching eggs, as previously reported. In this study, the morphological changes observed by scanning electron microscopy and the irritant response identified in the HET-CAM assay, as well as ECD in embryonic tracheas caused by GA, indicate that the undesirable effects of treatments with EOs are primarily attributable to GA rather than to the EOs themselves. These findings suggest that the solvent system’s current concentration may limit the safe large-scale application of EOs when diluted in alcohol under the tested conditions. Thus, the priority evaluation of using lower concentrations of GA, in addition to analyzing alternative non-alcoholic vehicles such as emulsifiers, emulsion systems, or nanoemulsions compatible with the process, considering bacterial contamination, gas exchange, and ensuring the quality and safety of embryonic development, may be a promising path to minimize the toxicity induced by the solvent at the concentration currently employed. The priority for experiments focused on the use of lower concentrations of GA is justified because alcohol has greater viability in achieving homogeneous dispersion, absence of residual film formation that could block shell pores, and faster surface drying, reducing the risk of immediate bacterial recontamination.

The EOs, despite their very distinct chemical compositions, showed individual effects with minimal differences among them when applied to hatching eggs. A summary of these effects is presented in Table 4.

## 3. Materials and Methods

### 3.1. Characteristics of Essential Oils (EOs)

The EOs used in this study were purchased and stored in strict accordance with the manufacturers’ recommendations (BioEssência^®^, São Paulo, Brazil), ensuring the integrity of their properties. The main characteristics of these oils are detailed in Table 5.

### 3.2. In Vitro Test of the Antibacterial Activity of Essential Oils (EOs)

The disk diffusion assay [56] using both pure and diluted EOs was previously employed as a criterion to determine the concentration of EO-based sanitizers. *Staphylococcus aureus* and *Escherichia coli* (*S*. *aureus*; *E*. *coli*; 100 μL; optical density: 0.5 McFarland; American Type Culture Collection, Manassas, VA, USA) were inoculated onto Mueller–Hinton agar (Difco, BD, Sparks, MD, USA). Sterile disks (6 mm in diameter; Laborclin, Pinhais, Paraná, Brazil) were impregnated with 10 μL of pure or diluted EOs of ZOEO, CFEO, and ROEO in 0.5% Tween 80. Twenty concentrations ranging from 2 to 0.1% (*v*/*v*), corresponding to 20 to 1 µL/mL, were tested. Control disks containing the antibiotic (azithromycin, 15 μg; Laborclin, Pinhais, Paraná, Brazil) and disks impregnated solely with 10 μL of 0.5% Tween 80 were placed on the surface of pre-inoculated Mueller–Hinton agar (Difco, BD, Sparks, MD, USA) and incubated at 36 °C for 24 h. The inhibition zones were observed after incubation. The lowest concentration of each EO, determined by the disk diffusion method and capable of simultaneously inhibiting the growth of both bacterial species tested, was recorded in this study. As *E. coli* and *S. aureus* are bacteria associated with the Gram-negative and Gram-positive microbiota contaminating eggshells, respectively, and serve as indicators of hygiene and bacteriological safety [45], these species were selected for the in vitro assays. The aim was to evaluate the antibacterial efficacy of the tested EOs and to obtain an initial estimate of their potential in vivo inhibitory effect. These preliminary results were crucial for planning the subsequent sanitization steps, as they allowed the determination of the optimal concentrations of the EO–based sanitizing agents. Table 6 presents the results obtained in this assay.

### 3.3. Poultry Sanitizers and Applications

**Figure 6:**
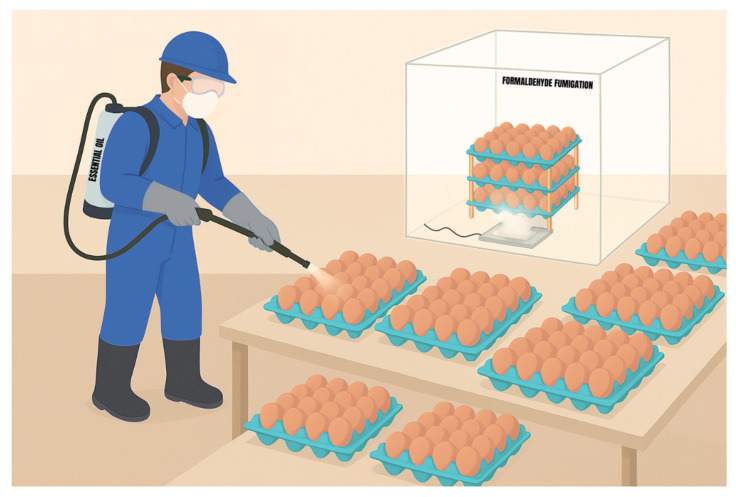
Illustration of the sanitization of hatching eggs with essential oils (EOs) and formaldehyde (FA).

Sanitizing solutions containing ZOEO, CFEO, and ROEO were prepared through controlled dilutions in 93.8% GA. The precise quantities of each EO were calculated based on the results of the disk diffusion assay, corresponding to concentrations of 0.9, 0.6, and 1.1%, respectively. These were the lowest concentrations of each oil that inhibited the growth of both *S*. *aureus* and *E. coli* (Table 6). The liquid solutions were applied using a sprayer, corresponding to approximately 1.5 mL per egg. During spraying, the eggs were rotated after each application to prevent layer overlap and ensure complete and uniform coverage of the entire eggshell surface. Fumigated eggs were exposed to fumigation inside a fumigation chamber using paraformaldehyde at 91% (Ercros, Barcelona, Spain). The compound was placed on a heated plate, which promoted the release of gaseous FA for a period of 15 min. The solutions were applied to the eggs after collection (20–50 min) (Figure 6). Table 7 provides some details of the tested protocols. The number of eggs used per treatment was defined in collaboration with the commercial hatchery, considering the routine availability of the setter and the sample sizes adopted in the literature [57]. The quality of the eggs used in the study was within the normal and excellent ranges for fresh eggs, as reported by the USDA [58] and Mineki and Kobayashi [59], with Haugh unit and yolk index values of 85.34 ± 4.04 and 0.41 ± 0.03, respectively.

### 3.4. Microbiological Analysis of Eggshell

The TAMB and ENT counts from the eggshell washing solutions were performed according to the methodology described by Vale et al. [14]. For each treatment, six different washing solutions were prepared. From each solution and its serial dilutions, 0.1 mL was pipetted onto the surface of Petri dishes containing count agar (Laborclin, Paraná, Brazil) and violet red bile glucose agar (Laborclin, Paraná, Brazil). The plates were incubated at 36 °C for 48 h. The CFUs were counted, and the results were log_10_ transformed.

### 3.5. Scanning Microscopy Analysis of Eggshell

After being prepared for microstructure analysis [60], the eggshell samples (eight/treatment) were subjected to metallization and examined using a JEOL JSM-7001F scanning electron microscope (Jeol Ltd., Akishima, Tokyo, Japan) at a standard magnification of up to ×4000. The images were subjected to multidimensional analysis, and using quantitative methodologies, morphological, textural, and structural alterations were recorded.

### 3.6. Incubation and Hatching

After 24 h of storage at the farm under controlled conditions of approximately 20 °C and 50% relative humidity, sanitized and non-sanitized eggs from 51-week-old Cobb broiler breeders were transported under refrigeration to a commercial hatchery. At the hatchery, the eggs underwent an additional 24 h storage period under the same conditions. Subsequently, they were incubated in a multi-stage setter with a capacity of 120,000 eggs (Avicomave, São Paulo, Brazil), where they remained for 18 days and were turned continuously every hour. On the 19th day of incubation, the eggs were transferred to a commercial hatcher with a capacity of 128,000 eggs (Avicomave, São Paulo, Brazil), where they remained for an additional 3 days. During the incubation period, the temperature was maintained between 37.5 °C and 38.0 °C, whereas in the hatcher, it was stable between 36.5 °C and 36.9 °C. In both stages, the relative humidity was consistently maintained at approximately 50%. To ensure that no interference could compromise the evaluation of the sanitizers, FA evaporation was avoided in both the setter and the hatcher. Additional information regarding incubation management is provided in Table 8.

### 3.7. Microbiological Analysis of the Yolk Sac

The TAMB and ENT counts from the yolk sac solutions were also performed [62]. For each treatment, three different yolk sac solutions were prepared (1 g of sample + 9 mL of 0.1% peptone saline solution; each mixture consisted of a mix of yolk sacs from two embryos). From each solution and its serial dilutions, 0.1 mL was pipetted onto the surface of Petri dishes containing count agar (Laborclin, Paraná, Brazil) and violet red bile glucose agar (Laborclin, Paraná, Brazil). The plates were incubated at 36 °C for 48 h. The colonies were counted, and the results were log_10_ transformed.

### 3.8. Histological Analysis of the Trachea

Embryos were euthanized by cervical dislocation, and their tracheae (six/treatment) were promptly collected and fixed in 10% formalin solution (pH 7.0), embedded in paraffin, and stained with hematoxylin and eosin for histopathological analysis. The methodology followed the protocol described by Oliveira et al. [15], which was adapted from Hayretdağ and Kolankaya [50].

### 3.9. Micronuclei Tests

Micronuclei, also known as Howell–Jolly bodies, were originally identified and described in erythrocytes. Micronuclei can arise through three main mechanisms: (1) acentric chromosomal fragments, (2) acentric chromatid fragments, or (3) whole chromosomes that fail to be incorporated into daughter nuclei during mitosis due to improper attachment to the mitotic spindle during anaphase segregation [63]. The micronucleus assay is a widely used biomarker to assess the potential effects of xenobiotics. This test has recently been investigated in birds by Souto et al. [64], Baesse et al. [65], and Gonçalves et al. [66]. Mutagenic and clastogenic effects typically arise following exposure to genotoxic agents [67]. Accordingly, the micronucleus assay is considered an effective cytogenetic endpoint for evaluating chromosomal damage induced by mutagens and carcinogens [68].

The micronucleus test was performed using blood samples collected from six chicks per treatment group immediately after hatching, following the protocols described by Souto et al. [64]. Additionally, six more chicks originating from eggs incubated in a different setter, where routine FA sanitization was applied during the incubation period, were also evaluated to investigate the potential effects of FA exposure during incubation (FA-IN). Blood samples were collected by puncturing the metatarsal vein using a 26G insulin needle (1 mL/U100), and one drop of blood was placed directly onto a glass slide. Blood smears were prepared on two slides per individual, which were fixed with absolute methanol for 10 min and subsequently stained with 5% Giemsa solution for another 10 min. The stained slides were analyzed under a light microscope (Zeiss Primo Star, Jena, Germany) at 400× magnification. For each smear, 1000 erythrocytes were counted. Upon reaching this number, the occurrence of cells with micronuclei and/or nuclear abnormalities, including binucleated, notched, lobed, blebbed, kidney-shaped, anucleated, pyknotic, and apoptotic cells, was recorded to assess the frequency of these alterations [69].

### 3.10. Hen’s Egg Test Chorioallantoic Membrane (HET-CAM) of Sanitizers

A total of 45 fertile eggs from Pescoço Pelado Vermelho broiler breeders were removed on day 10 of incubation from a commercial multistage setter with a capacity of approximately 115,000 eggs (Coopermaq, Santa Catarina, Brazil). The setter operated at 37.5–38.0 °C with 50–60% relative humidity and automatically turned every hour. After the eggs were removed from the setter, the eggshell and its membranes (moistened with 0.9% saline solution) located in the air chamber were carefully removed with tweezers, exposing the CAM. A volume of 200 μL of each EO, diluted or undiluted in GA, as well as GA or FA (diluted in distilled water), was applied directly onto the CAM. All concentrations were the same as those used on the eggshell. A control group, without the application of any sanitizing agent, was also evaluated. Reactions occurring on the membrane were monitored for 5 min and documented through photographs to identify hemorrhage, coagulation, and blood vessel lysis [70].

### 3.11. Statistical Analysis

Statistical analyses were performed using GraphPad Prism 5 (https://www.graphpad.com) or SAS software version 9.4 (SAS Institute Inc., Cary, NC, USA), with the significance level set at *p* < 0.05. Data were compared among experimental groups using analysis of variance followed by Tukey’s test (PROC GLM), or the Kruskal–Wallis test (PROC NPAR1WAY) for normally and non-normally distributed data, respectively. The following variables showed normal distribution: EWBS (F(5) = 0.36, *p* = 0.8704), EWDT (F(5) = 1.67, *p* = 0.1932), EWL (F(5) = 1.94, *p* = 0.1374), CW (F(5) = 3.82, *p* = 0.0156), HI (F(5) = 1.29, *p* = 0.3124), EED (F(5) = 0.90, *p* = 0.5051), MED (F(5) = 0.49, *p* = 0.7825), LED (F(5) = 0.40, *p* = 0.8420), CE (F(5) = 1.64, *p* = 0.2005), and yolk sac TAMB (F(5) = 4.64, *p* = 0.0137). The non-normally distributed variables were the eggshell TAMB (F(5) = 26.54, *p* < 0.0001) and CQS (χ^2^(5) = 9.63, *p* = 0.0863).

## 4. Conclusions

ZOEO, CFEO, and ROEO were shown to have sanitary and safety compatibility for the commercial sanitization of hatching eggs, contributing to the maintenance of high productivity rates in hatcheries with performance comparable to that of FA. However, future economic analyses should be conducted to verify their financial compatibility. These compounds did not cause severe damage to the eggshell and significantly reduced the bacterial load on the eggshell surface and in the yolk sac, which is essential for minimizing the risk of contamination and infection. Furthermore, the use of EOs resulted in embryos without tracheal issues and in chicks with higher hatch weights than those from eggs treated with FA. Considering their potential residual effects, another important point is that significant genetic damage is unlikely to occur in chicks hatched from eggs sanitized with OEs. However, further studies are needed to confirm this hypothesis, through improvements in the applied methodology or using more specific and sensitive evaluation methods. Finally, the EOs tested in this study, when diluted in GA, should be applied exclusively to the eggshell surface, following the tested concentrations and using appropriate protective equipment such as gloves, masks, and lab coats. It is also recommended that future studies evaluate these protocols using lower concentrations of GA to determine whether potential adverse effects associated with its application to hatching eggs can be minimized.

## Figures and Tables

**Table 1:** Bacterial counts on eggshells and embryonic yolk sacs of sanitized and non-sanitized eggs.

**Sanitizer**	**Eggshells**	
	**TAMB**	**ENT**
	**(log_10_ CFU/mL)**	
Control	1.60 ± 0.30 ^a^	<1
GA	1.13 ± 0.25 ^a^	<1
FA	<1 ^b^	<1
ZOEO	<1 ^b^	<1
CFEO	<1 ^b^	<1
ROEO	<1 ^b^	<1
*p* value	<0.0001	
**Sanitizer**	**Yolk Sacs**	
	**TAMB**	**ENT**
	**(log_10_ CFU/mL)**	
Control	2.58 ± 0.39 ^a^	<1
GA	1.66 ± 0.48 ^ab^	<1
FA	1.99 ± 0.10 ^ab^	<1
ZOEO	1.06 ± 0.20 ^b^	<1
CFEO	1.13 ± 0.99 ^b^	<1
ROEO	1.00 ± 0.35 ^b^	<1
*p* value	0.0137	

^a,b^ Different letters in the same column mean significant differences using the Tukey test (*p* < 0.05). Grain alcohol—GA; formaldehyde—FA; *Zingiber officinale* essential oil—ZOEO; *Cymbopogon flexuosus* essential oil—CFEO; *Rosmarinus officinalis* essential oil—ROEO; total aerobic mesophilic bacteria—TAMB; Enterobacteriaceae—ENT.

**Table 2:** Results of the incubation of sanitized and non-sanitized eggs.

Sanitizer	EWBS (g)	EWDT (g)	EWL (%)	CW (g)	HI (%)
Control	66.25 ± 1.48 ^a^	57.71 ± 0.98 ^a^	12.87 ± 0.95 ^a^	45.97 ± 0.67 ^ab^	90.36 ± 4.88 ^a^
GA	66.98 ± 0.43 ^a^	59.21 ± 037 ^a^	11.59 ± 0.18 ^a^	46.28 ± 0.92 ^a^	92.02 ± 3.30 ^a^
FA	66.69 ± 0.43 ^a^	58.14 ± 0.57 ^a^	12.82 ± 0.61 ^a^	44.94 ± 0.42 ^b^	90.29 ± 2.28 ^a^
ZOEO	66.56 ± 0.63 ^a^	58.42 ± 0.75 ^a^	12.24 ± 0.61 ^a^	46.32 ± 0.33 ^a^	93.48 ± 3.60 ^a^
CFEO	66.26 ± 1.67 ^a^	57.79 ± 1.38 ^a^	12.77 ± 1.07 ^a^	46.50 ± 0.57 ^a^	93.16 ± 1.06 ^a^
ROEO	66.24 ± 0.61 ^a^	58.07 ± 0.68 ^a^	12.34 ± 0.43 ^a^	46.14 ± 0.31 ^ab^	94.66 ± 2.04 ^a^
*p* value	0.8704	0.1932	0.1374	0.0156	0.3124

^a,b^ Different letters in the same column mean significant differences using the Tukey test (*p* < 0.05). Grain alcohol—GA; formaldehyde—FA; *Zingiber officinale* essential oil—ZOEO; *Cymbopogon flexuosus* essential oil—CFEO; *Rosmarinus officinalis* essential oil—ROEO; EWBS, egg weight before setting; EWDT, egg weight during transfer; EWL, egg weight loss; CW, chick weight; HI, hatchability.

**Table 3:** Analysis of embryonic tracheal tissues from sanitized and non-sanitized eggs ^1^.

Sanitizer	Tracheal Lesion			
	ECN	GCH	LI	ECD
Control	−	−	−	−
GA	−	−	−	+
FA	−	+	+	++
ZOEO	−	−	−	−
CFEO	−	−	−	−
ROEO	−	−	−	−

^1^ The data are presented in the following intensity categories: absent (−), mild (+), and moderate (++). Grain alcohol—GA; formaldehyde—FA; *Zingiber officinale* essential oil—ZOEO; *Cymbopogon flexuosus* essential oil—CFEO; *Rosmarinus officinalis* essential oil—ROEO; Epithelial cell necrosis—ECN; Goblet cell hyperplasia—GCH; Lymphocytic inflammation—LI; Epithelial cell degeneration—ECD. The results are the means obtained by measuring samples of six embryos.

**Table 4:** Main effects of each essential oil (EO) on hatching egg sanitization.

EO Treatment	Main Effects
ZOEO	Reduced TAMB on eggshells and yolk sacs. Did not cause severe effects on eggshells. Promoted high HI rates. Did not cause embryonic tracheal effects. Did not cause significant genotoxic effects in chicks. Produced heavier chicks compared to FA. Classified as non-irritant in its pure form.
CFEO	Reduced TAMB on eggshells and yolk sacs. Did not cause severe effects on eggshells. Promoted high HI rates. Did not cause embryonic tracheal effects. Did not cause significant genotoxic effects in chicks. Produced heavier chicks compared to FA. Classified as a mild irritant in its pure form.
ROEO	Reduced TAMB on eggshells and yolk sacs. Better preserved eggshell integrity. Promoted high HI rates. Did not cause embryonic tracheal effects. Did not cause significant genotoxic effects in chicks. No significant changes in CW. Classified as non-irritant in its pure form.

*Zingiber officinale* essential oil—ZOEO; *Cymbopogon flexuosus* essential oil—CFEO; *Rosmarinus officinalis* essential oil—ROEO; total aerobic mesophilic bacteria—TAMB; HI, hatchability; CW, chick weight.

**Table 5:** Characteristics of commercially acquired essential oils (EOs) from *Zingiber officinale* (ZOEO), *Cymbopogon flexuosus* (CFEO), and *Rosmarinus officinalis* (ROEO).

EO	Extraction Method	Density (20 °C)	Refraction Index (20 °C)	Main Chemical Compound
ZOEO	Steam distillation of the rhizome	0.873	1.486	α-Zingiberene − 33.92%
CFEO	Steam distillation of the leaves	0.888	1.483	Geranial − 49.05%
ROEO	Steam distillation of the leaves	0.899	1.466	α-Pineno − 23.03%

**Table 6:** Results of the disk diffusion assay.

Bacteria	AZ	T80	ZOEO	CFEO	ROEO
	ZOI (mm) ± SD (*n* = 3)		LEC (μL/mL) ± SD (*n* = 3)		
*S. aureus*	21.15 ± 1.28	no zone	9 ± 0.44	6 ± 0.16	11 ± 0.35
*E. coli*	25.71 ± 0.93	no zone	8 ± 0.15	4 ± 0.27	10 ± 0.54

Azithromycin—AZ; tween 80—T80; *Zingiber officinale* essential oil—ZOEO; *Cymbopogon flexuosus* essential oil—CFEO; *Rosmarinus officinalis* essential oil—ROEO; zone of inhibition—ZOI; standard deviation—SD; lowest effective concentration—LEC: essential oil concentration ranging from 20 to 1 μL/mL.

**Table 7:** Details of the application of sanitizers to eggs.

Sanitizer	Concentration	Application Method	Sanitizer Amount (Approx.)	Sanitizer Temperature	Room Temperature	Number of Eggs
Control	.	.	.	23–24 °C	23–24 °C	350
GA	93.8%	Spraying	500 mL	20–21 °C	23–24 °C	350
FA	5 g/m^3^	Fumigation	5 g	24–26 °C	23–24 °C	350
ZOEO	0.9%	Spraying	500 mL	20–21 °C	23–24 °C	350
CFEO	0.6%	Spraying	500 mL	20–21 °C	23–24 °C	350
ROEO	1.1%	Spraying	500 mL	20–21 °C	23–24 °C	350

Grain alcohol—GA; formaldehyde—FA; *Zingiber officinale* essential oil—ZOEO; *Cymbopogon flexuosus* essential oil—CFEO; *Rosmarinus officinalis* essential oil—ROEO.

**Table 8:** Incubation management during the experimental period.

Incubation Day	Activity
0	Identification of the incubation trays. All eggs were weighed. The eggs were distributed in the trays (84 eggs/tray), and the trays with the different treatments were positioned in the incubation trolley in such a way as to ensure that each treatment was represented in the highest, lowest, central, above-middle, and below-middle positions.
18	All eggs were weighed again. Fertile eggs were removed from the setter for trachea extraction and bacterial count of the embryo’s yolk sac.
21–22	Counting and weighing of all hatched chicks. Measurement of the quality of 40 chicks per treatment [61]. Blood collection from newly hatched chicks. Embryo diagnostics of unhatched eggs: - Identification of infertile eggs. - Dead period: early, mid, or late. - Contaminated eggs.
Formulas used to calculate incubation parameters: Egg weight loss (%) = [(initial egg weight − egg weight measured on the transfer day)/initial egg weight] × 100. Hatchability (%) = (number of hatched chicks/number of fertile eggs) × 100. Early dead (%) = (number of dead embryos on days 0–7 of incubation/number of fertile eggs) × 100. Mid dead (%) = (number of dead embryos on days 8–18 of incubation/number of fertile eggs) × 100. Late dead (%) = (number of dead embryos on days 19–21 of incubation/number of fertile eggs) × 100. Contaminated eggs (%) = (number of contaminated eggs/number of fertile eggs) × 100.	

## Data Availability

The data is contained within the article.
